# Table of Societies

**Published:** 1871-01

**Authors:** 


### TABLE OF SOCIETIES.

For several years past we have published a list of all the Dental Societies in the United States, the time and place of their meetings, and the names of the principal officers. This has been done at an expense of $75 to $100 per annum by the publisher,—a *gratuity* to the profession. With the March number we propose a change in this particular, viz: that all Societies that wish a place in this List make an annual contribution of $5 to assist in defraying the expense of its publication. No claim is here made upon any Society either for the past or the future, but any one making the contribution will have a place in the list. This is the best and indeed the only systematic notice of the meetings of Societies that has ever been given. This work was entered upon in the outstart, with an entire willingness and a desire to encourage the organization of local societies, and if possible stimulate those already in existence to greater activity. We shall be pleased to receive a corrected notice from every Society wishing a place in this List.

